# Towards Animal-Free Toxicology: Establishment of Two Larval Brown Trout Cell Lines for Environmental Risk Assessment

**DOI:** 10.3390/toxics13080696

**Published:** 2025-08-20

**Authors:** Bianka Grunow, Valeria Di Leonardo, Katrin Tönißen

**Affiliations:** Work Group “Fish Growth Physiology”, Research Institute for Farm Animal Biology (FBN), 18196 Dummerstorf, Germany; di-leonardo@fbn-dummerstorf.de (V.D.L.); toenissen@fbn-dummerstorf.de (K.T.)

**Keywords:** water pollution, in vitro, fish cell line, xCelligence, fish toxicity, environmental stress

## Abstract

Advances in cell culture technology have led to fish cell lines being used as cost-effective, reproducible, and ethically favourable instruments in ecotoxicology. The development of new lines contributes to reducing animal experiments and improves model diversity. The brown trout (*Salmo trutta*), an important bioindicator due to its sensitivity to pollutants, is still underrepresented in cell culture systems. In this study, two novel larvae-derived cell lines, STRlar1 and STRlar2, were established and maintained for over 40 passages. Although derived from sibling larvae of the same parental strain, the two lines showed marked differences in growth dynamics and toxicological responses to ethanol, dimethyl sulfoxide, isopropanol, and acetone. STRlar2 showed greater sensitivity to all chemicals tested, while STRlar1 exhibited longer proliferation and higher impedance, suggesting stronger substrate adhesion. These differences emphasise the importance of cellular heterogeneity, even in closely related lines. Our results underline the need to carefully validate new in vitro models and caution against relying on single cell lines. To improve the robustness and reliability of ecotoxicological assessments, we recommend the use of multiple independently derived lines that better reflect biological variability and reflect in vivo complexity.

## 1. Introduction

The brown trout *Salmo trutta* (family Salmonidae) is a species native to Europe and Western Asia. It has been widely introduced to other parts of the world for consumption and recreational fishery, including North America, Australia, and New Zealand, where it has successfully established populations in numerous freshwater habitats, outcompeting native species [1,2,3]. For this reason, the International Union for Conservation of Nature (IUCN) listed this species in the 100 of the World’s Worst Invasive Alien Species list [4]. Brown trout are highly adaptable, inhabiting a wide range of environments—from small streams and rivers to large lakes. Some populations are even anadromous, the so-called sea trout [5].

Despite recent improvements in population status in Europe—reflected by the IUCN’s 2022 reclassification of brown trout from ‘Near Threatened’ to ‘Least Concern’ [6]—local populations still face significant threats. These include habitat degradation, pollution, overfishing, invasive species, and the impacts of climate change, particularly rising temperatures that are expected to strongly affect cold-water species like the brown trout [7].

Brown trout are not only ecologically and economically significant but also serve as important bioindicator species in ecotoxicology due to their sensitivity to environmental pollutants. Research on brown trout has provided valuable insights into the effects of environmental pollutants on aquatic ecosystems as well as fish physiology and health [8,9,10,11]. In view of the increasing number of chemical substances regulated by legislation such as the European REACH Regulation (Registration, Evaluation, Authorization and Restriction of Chemicals) and the increasing presence of chemicals in everyday life, there is a growing need to assess their bioactivity. In this context, in vitro models have become indispensable. The use of fish cell lines in ecotoxicological research offers a promising alternative to tests on live animals [12,13,14]. High correlations between in vitro and in vivo results have been reported [15], supporting their relevance for regulatory purposes. A milestone was reached with the introduction of the ISO 21115:2019 standard [16], which allows the use of the rainbow trout (*Oncorhynchus mykiss*) gill cell line RTgill-W1 originating from juvenile fish [17] according to OECD guidelines. Despite these advances, tests still often rely on live fish larvae [18], particularly due to the lack of cell lines derived from early stages of development [19].

Trout larvae remain essential models in ecotoxicology, as their high sensitivity during early development allows for the detection of subtle or delayed toxic effects that may be missed in adult fish. Early life-stage tests using trout are commonly applied to assess acute and sublethal toxicity, endocrine disruption, and developmental abnormalities caused by various pollutants [20,21,22]. These tests offer both ecological relevance and a partial ethical advantage, as early larval stages are often exempt from animal welfare regulations. Recent developments in cell culture technology highlight the need for a broader range of fish cell lines, particularly from larvae and diverse species, to better represent biological variability and reduce the use of live animals [23,24,25,26,27].

Fish cell lines from 211 species and hybrids are listed on the Cellosaurus database, with rainbow trout, goldfish (*Carassius auratus*) and zebrafish (*Danio rerio*) as the most represented species [19,28]. Unique properties of species and organ or tissue are crucial when analysing the effects and mode of action of a substance [29,30]. In fact, the RTgill-W1 mentioned above is crucial for assessing pollutants that impair gill respiration and osmoregulation [26], while the RTG-2 cell line from rainbow trout gonads is useful for studying chemicals interfering with endocrine systems [12,31]. The RTL-W1, derived from rainbow trout liver, is pivotal for the metabolism of toxicants [32].

Larval cell lines offer unique advantages: they reflect the heightened vulnerability of developing organisms and allow toxicity testing without the confounding effects of metabolisation [33]. This is especially relevant since many compounds may act differently before metabolic pathways are fully developed. Moreover, larval-derived cultures can yield multipotent stem-like cells from various tissues, capturing a broader physiological context [34,35,36]. Such models have the potential to refine developmental toxicity testing and further reduce the need for animal use [37,38]. The growing interest in embryonic and larval models reflects not only scientific and ethical progress but also the regulatory gap in protecting early stages of non-mammalian species [39].

Therefore, the aim of the present study was twofold: first, to establish two brown trout cell lines from whole larval tissue and characterise their growth; second, to expose these cell lines to various ecotoxicological solvents and concentrations commonly used in industry in order to analyse and compare the effects on the cells and intraspecific diversity. To our knowledge, this is the first study in which two cell lines from brown trout larvae have been established and used for toxicological purposes, creating a novel platform for the animal-free screening of environmental hazardous substances for risk assessment.

## 2. Materials and Methods

### 2.1. Ethic Statement

This study did not involve any animal experiments (Animal Welfare Directive 2010/63/EU and German TierSchG § 4(3)). Nevertheless, this study followed international, national, and institutional guidelines for the treatment and killing of animals and complied with Directive 2010/63/EU and the German Animal Welfare Act [§ 4(3) TierSchG]. Within this study, unhatched *Salmo trutta* in the eye point stage were used. Unfortunately, there are no specific regulations for fish larvae and brown trout within the Directive 2010/63/EU [40].

To euthanise the larvae within the eggs as gently and quickly as possible, the beaker containing the eggs was placed on ice to lower the water temperature, a method known as temperature-induced hypothermia, which reduces the nervous activity and metabolism of the fish. This technique temporarily slows down the physiological processes of the fish (larvae), making them less active and reduces the stress. After a 10 min incubation period, larvae were isolated from their eggshells and immediately euthanised by performing a neck break followed by decapitation using forceps.

### 2.2. Cell Isolation, Cell Culture, and Cryopreservation

Brown trout (*Salmo trutta*) eggs in the eye point stage were obtained from the Fisher Station of Hohen Sprenz in Mecklenburg-Vorpommern, Germany. The fish larvae were euthanised as described above. Before cell isolation, the larval tissue was thoroughly washed three times in 1x PBS (Dulbecco’s Phosphate-Buffered Saline; PAN-Biotech, Aidenbach, Germany). Subsequently, the larval tissue was minced with scissors while being digested for one minute in a 0.1% trypsin/EDTA solution (Gibco, Paisley, UK) following the protocols of Grunow et al. (2011) and Kaya et al. (2022) [41,42]. The digestion was halted by adding double the amount of L-15 medium (Leibovitz-15 Medium, Gibco, Paisley, UK) supplemented with 20% FBS (foetal bovine serum, PAN-Biotech) and 1% (*v*/*v*) P/S (penicillin/streptomycin, Gibco Life Technologies). After centrifugation at 130× *g* for 5 min, the cells were resuspended in cell culture medium supplemented with additional antibiotics (Gentamycin: 0.1 mg/mL; Gibco, Paisley, UK and Kanamycin: 0.5 mg/mL; Grand Island, NY, USA) and an antimycotic agent (Amphotericin: 250 μg/mL, Gibco, Paisley, UK). Cells from each larva were seeded into a 6-well plate (TPP, Techno Plastic Products AG, Trasadingen, Switzerland) and incubated at 22 °C under normoxic conditions in an NU-8631E incubator (IBS Tecnomara, Fernwald, Germany). The primary cell culture was maintained undisturbed for 48 h. Afterwards, washing with PBS and medium exchange was performed. During the first two weeks, the medium was changed every two days, and the above-mentioned additional antibiotics were added to the cell culture medium. Later on, medium exchange was performed every third or fourth day without additional antibiotics, except for P/S. Due to insufficient growth, the cells of more larvae (vessels) were digested with 0.1% trypsin/EDTA solution and seeded in a T25 flask (TPP, Techno Plastic Products AG) after 31 days of primary culture. Afterwards, cells began to proliferate more rapidly, and upon reaching confluence, the cells were sub-cultured at a 1:2 ratio using 0.1% trypsin/EDTA in PBS (phosphate-buffered saline; PAN-Biotech, Aidenbach, Germany). After detachment, the cells were centrifuged at 130× *g* for 5 min. The resulting cell pellet was resuspended in fresh culture medium and seeded into new culture dishes. For the first 10 passages, the cells were continuously grown in L-15 medium supplemented with 20% FBS. Subsequently, the medium was changed to L-15 with 15% FBS for two passages, followed by the use of 10% serum additive for further cultivation from passage 12 onwards. The fish cell lines are called STRlar1 and STRlar2.

For cryopreservation, trypsinised cells were resuspended in ice-cold freezing medium composed of a 9:1 ratio of FBS to dimethyl sulfoxide (DMSO). The cell suspension was then transferred into a pre-cooled isopropanol freezing container and stored at −80 °C overnight or for up to two weeks. Subsequently, the vials were moved to a liquid nitrogen atmosphere for long-term storage. For reseeding, the frozen cells were quickly thawed in cell culture medium pre-warmed to room temperature, centrifuged at 130× *g* for 5 min, resuspended, and seeded into fresh culture medium. If frozen cells needed to be thawed for the experiments, cells were sub-cultured at least twice before being used in the experiments. STRlar1 and STRlar2 cells between passage 17 and 35 were used for all experiments.

### 2.3. Observation of Cell Viability

Cell morphology and attachment were examined using an inverted phase-contrast microscope (Motic^®^ AE2000, Wetzlar, Germany). Images were captured with Motic Images Plus 3.0 Software and further processed for brightness and contrast adjustments using Adobe Photoshop CC 2019 (Adobe Inc., San Jose, CA, USA). Throughout all passaging, freezing, and thawing procedures, the total cell count, viability percentage (determined by trypan blue staining), and cell size were measured using the EVE^TM^ Plus Automated Cell Counter (NanoEnTek, Gyeonggi-do, Korea). Data are presented as box–whisker plots. The group comparisons were performed using a two-tailed Student’s *t*-test. Significant differences were defined by *p* < 0.05 calculated in Graphpad Prism Version 10.5.0 (GraphPad Software Inc., now part of Dotmatics, Boston, MA, USA).

### 2.4. Comparative Analysis of Two STRlar Cell Lines

To obtain well-proliferating cell lines, it was necessary to combine cells from different larvae during the developmental phase of the cell line. Therefore, for the STRlar1 cell line, cells of five larvae were pooled to passage 1 (P1), and for the STRlar2 cell line, cells of twelve larvae were pooled together.

A comparative analysis of STRlar1 and STlar2 cells was conducted to evaluate the impact of different serum concentrations on cell viability and size. Cells were cultured in T25, T75, and T150 cell culture flasks (TPP, Switzerland) with either 10% or 20% FBS. Data for cells grown with 20% FBS were collected from passages 3 to 9 (*n* = 7–8 for each cell line), while those cultivated with 10% FBS (*n* = 11–14 for each cell line) were analysed from passages 13 and onwards.

Early passaging resulted in reduced growth rates or even cell death, indicating the necessity of determining the optimal timing for passaging. Therefore, cell behaviour was observed over a period of 4 days to assess their growth characteristics, and then again on days 10 to 13, the day of passaging. Cell number, cell size, and viability were measured, and observations were made using phase-contrast microscopy. Experiments were repeated twice with three independent trials (*n* = 6) between passage 20 and 33. Data are presented as box–whisker plots and the group comparisons were performed via one-way ANOVA with Tukey’s test. Significant differences were defined by *p* < 0.05 calculated in Graphpad Prism Version 10.5.0 (GraphPad Software Inc., now part of Dotmatics, Boston, MA, USA).

### 2.5. Ecotoxicological Studies

Firstly, the optimal cell seeding concentration was analysed using xCelligence RTCA SP instrument (ACEA Biosciences Inc. now part of Agilent Technologies, Santa Clara, CA, USA), operating in an NU-8631E incubator (Ibs Tecnomara, Fernwald, Germany) with starting cell concentrations of 5000 cells/cm^2^, 10,000 cells/cm^2^, and 15,000 cells/cm^2^ (*n* = 6) in E-Plate 96 for xCELLigence RTCA SP/MP (Agilent Technologies, San Diego, CA, USA). For all further experiments, a seeding density of 5000 cells/cm^2^ was used to enable cell observation over a 7-day period. Higher concentrations led to stagnation occurring too early, which interfered with the continuous monitoring and analysis of cell behaviour.

In the ecotoxicological experiments, the cell status was monitored in real time over a 7-day period at 15 min intervals, measuring the cell impedance of STRlar1 cells at passages 17 and 21, as well as STRlar2 cells at passages 30 and 33. STRlar1 and STRlar2 cells were seeded, and after 24 h for cell attachment and adaptation, a medium exchange was conducted in the control wells, while in the experimental wells, various chemical solvents commonly used in industry were added at different concentrations prepared in culture medium covering a wide range of industrial applications, as described in the study by Grunow et al. (2021) [43]. The chemicals used were as follows: I. CH3CH2OH (ethanol): 4.0%, 2.0%, 1.5%, 1.0%, 0.5%, 0.25%; II. (CH3)2CHOH (2-Propanol—Isopropanol): 2.0%, 1.0%, 0.5%, 0.25%, 0.125%; III. (CH3)2SO (DMSO): 20%, 10%, 5%, 2.5% 1.0%, 0.5%; and IV: C3H6O (acetone): 2.0%, 1.0%, 0.5%, 0.1%, 0.01%. The concentrations of substances were selected based on the previously conducted study on larval cell lines derived from maraena whitefish (*Coregonus maraena*) and Atlantic sturgeon (*Acipenser oxyrinchus*) to ensure comparability [43]. All experiments were conducted in two independent runs, each with four replicates per treatment (*n* = 8). Growth differences between the control and chemically treated groups were calculated relative to the respective control values. In this study, three time periods were evaluated. Acute toxicity was determined as the average mortality (% mortality) observed during the first 3.5 h following chemical exposure (hours 24–27.5). This period coincided with a marked cellular response in the growth curves. Mid-term toxicity was assessed based on mean mortality values measured between hours 112 and 118, a phase in which cells in both control groups were still actively proliferating. Long-term toxicity was evaluated during the final phase of the experiment, using data from hours 159 to 165. At this point, STRlar1 cells had reached growth stagnation, while STRlar2 cells had entered a post-peak phase.

Single mean growth curves for each experimental trial were calculated from eight replicates for subsequent illustration and final analysis. For toxicological assessments, data were normalised to the time point of chemical addition (hour 24). Likewise, percentage changes in cell impedance were referenced to the impedance value immediately before chemical exposure to calculate mortality. All results are presented as mean ± SEM. Acute, medium-term and long-term toxicity was analysed via Kruskal–Wallis followed by Dunn’s test for multiple comparisons compared against the control. Significant differences were defined by *p* < 0.05 calculated in GraphPad Prism Version 10.5.0 (GraphPad Software Inc., now part of Dotmatics, Boston, MA, USA).

## 3. Results

### 3.1. Observation of Larval Brown Trout Cell Migration

**Figure 1:**
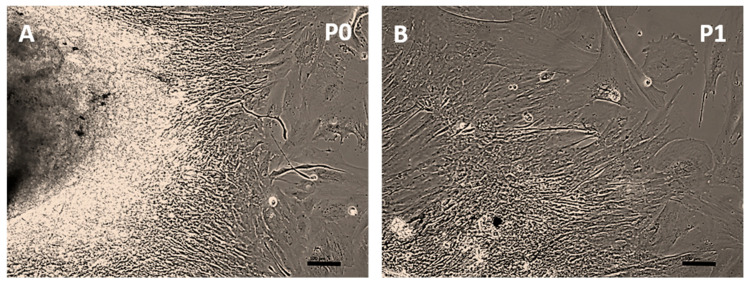
The culture process of STRlar cells after cell isolation. (**A**) Cells in passage P0 migrate out of tissue fragments. (**B**) In passage P1, areas of compact cell layers are still present. Cells display different morphologies. Scale: 100 µm.

Immediately after isolation, a suspension of cells and tissue fragments was obtained in cell culture. Within 48 h, these components adhered to the surface of the cell culture dishes, initiating cell outgrowth and proliferation from the attached tissues (Figure 1A). Non-adherent cells and debris were removed during the first medium exchange, performed two days post-isolation. In the first three passages, cells displayed a high degree of morphological diversity, with shapes ranging from spindle-like to round and extremely flattened (Figure 1A,B), a pattern observed in both cell lines.

**Figure 2:**
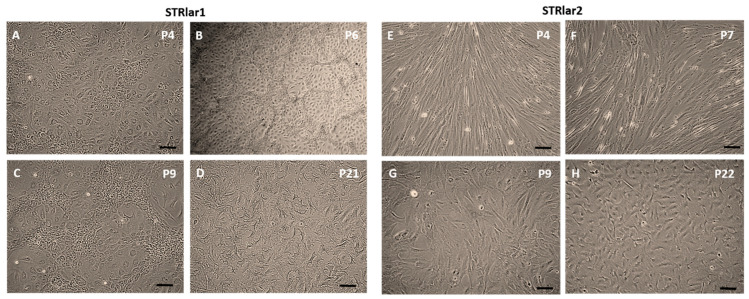
The culture process of STRlar1 and STRlar2 cells in different passage (P). In early passages cells were cultivated with 20% FBS (**A**,**B**,**E**,**F**). STRlar1 cells built a 3D network between P5 and P10 (**B**,**C**), which stopped when reducing FBS to 15% and from P13 onwards to 10% (**D**). In Strlar2 cells, no 3D network was visible (**E**–**H**). Scale: 100 µm.

With increasing passage number, cell morphology became more uniform, though distinct differences between the two cell lines persisted (Figure 2). STRlar1 cells appeared more round and compact (Figure 2A–C), while STRlar2 cells were more elongated (Figure 2E–G).

Between passages 5 and 10, STRlar1 cells formed a 3D network with a monolayer in the centre (Figure 2B,C). This 3D structure disappeared after reducing FBS to 15% at passage 10 and did not reoccur with a further reduction to 10% FBS from passage 13 onwards (Figure 2D). STRlar2 cells never formed a 3D network; instead, by passage 9, they became more compact and uniform, losing their elongated shape (Figure 2H).

**Figure 3:**
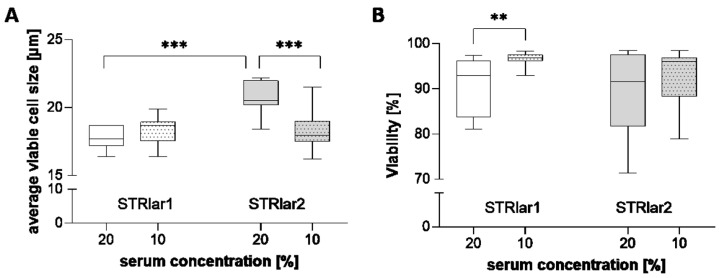
Overview of cell size (**A**) and viability (**B**) in STRlar1 (white boxes) and STRlar2 (grey boxes) cell lines in early passages (P3–P9, clear boxes) cultivated with 20% FBS and in later passages (>P13, dotted boxes) cultivated with 10% FBS. For statistical analysis a two-tailed Student’s *t*-test was performed within the cell line (10 vs. 20% FBS) and for a cell line comparison of the same serum concentration. Statistical significance (one-way ANOVA) as indicated: ** *p* ≤ 0.01, *** *p* ≤ 0.001 (*n* = 7–8 for 20% FBS and *n* = 11–14 for 10% FBS conditions; GraphPad Prism 10.5.0.).

In STRlar1, average cell size remained stable across early and late passages as well as under reduced serum conditions (Figure 3A). Both cell lines showed significantly higher and more stable viability in higher passages with 10% serum compared to passages 3–9 with 20% serum (Figure 3B).

Passage procedures, including freezing and thawing, did not negatively affect cell viability. After passaging, viability was 93.7 ± 1.6% (STRlar1) and 93.2 ± 1.6% (STRlar2), and after thawing, viability remained between 90 and 92% in both lines. Notably, both cell lines have been successfully maintained through over 40 passages, demonstrating their robustness and long-term stability in culture.

### 3.2. Cell Culture Handling

**Figure 4:**
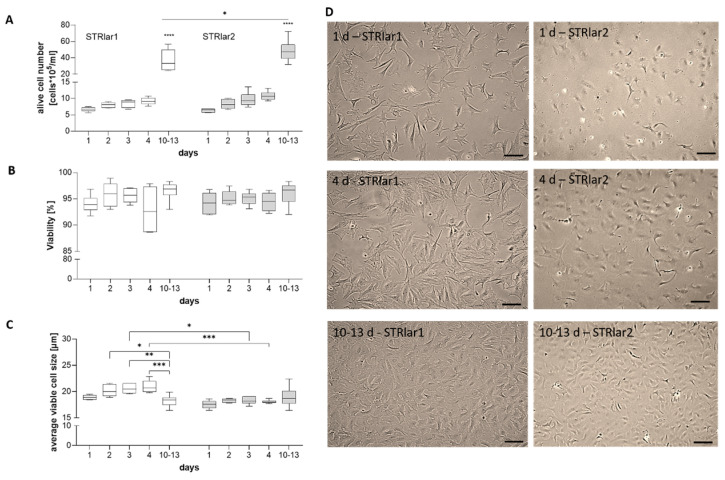
Cell observation at different time points of STRlar1 and STRlar2. Cell number (**A**), viability (**B**), cell size (**C**) and phase contrast pictures (**D**) were taken at day 1, 2, 3, 4 and 10–13 days after passaging. Scale: 100 µm. Statistical significance (one-way ANOVA) as indicated: * *p* < 0.05, ** *p* < 0.01, *** *p* < 0.001, **** *p* < 0.0001); *n* = 6 for each cell line; GraphPad Prism 10.5.0.).

Premature passaging of both cell lines led to reduced or halted proliferation. Optimal passaging occurred between days 10 and 13. Therefore, proliferation, viability, and cell size were monitored at multiple time points (Figure 4). Cell counts remained stable during the first four days, indicating minimal proliferation early on. A significant increase in proliferation was observed after 10 days for both STRlar lines, with STRlar2 showing higher cell numbers than STRlar1 at this point (Figure 4A). Cell viability stayed consistently high throughout the experiment without significant differences (Figure 4B). Cell size was stable during the first four days; however, STRlar1 cells showed a size reduction between days 10 and 13, resembling their size on day one. STRlar2 cells were generally smaller, with significant size differences compared to STRlar1 on days three and four (Figure 4C). Morphologically, STRlar1 cells displayed diverse shapes with lamellipodia and filopodia, whereas STRlar2 cells maintained a more compact, rounded to slightly branched morphology throughout (Figure 4D).

### 3.3. Cell Impedance and Response to Chemical Solvents

**Figure 5:**
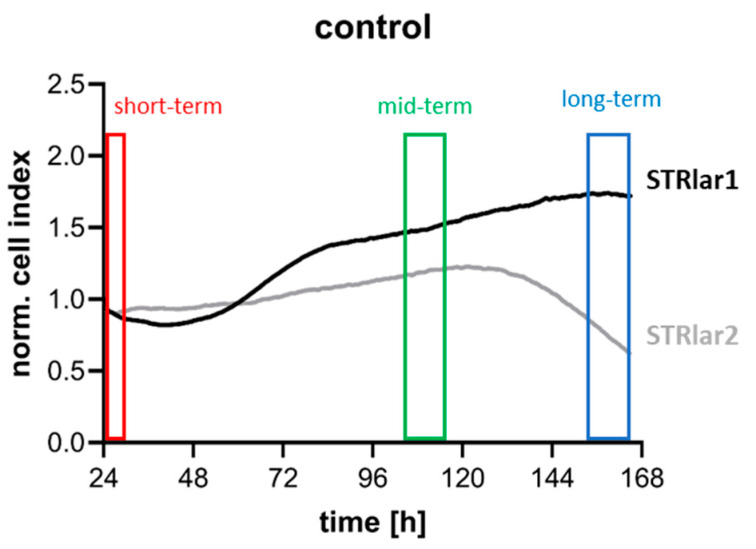
Cell index analysis of STRlar1 (black curve) and STRlar2 (grey curve) over a 6-day period. Following 24 h of incubation, a media exchange to chemical solvents was performed, marking the onset of the normalisation (index 1.0; *n* = 8 for each cell line). In this graph, time points for analysing chemical responses are highlighted, with the short-term reaction (red box), mid-term reaction (green box), and the long-term reaction (blue box). These reactions will be analysed in comparison to the control cells to assess the impact of medium exchange (short-term), the proliferation phase (mid-term), and the stagnation, respectively, post-peak phase of the cells.

The analyses revealed distinct variations in the impedance curve profiles, reflecting varied cellular behaviour between the two brown trout larval cell lines (Figure 5). The medium exchange performed 24 h after attachment resulted in a drop in cell impedance across all wells, likely due to the removal of cells with weaker adhesion. Despite this, both cell lines recovered quickly, showing an increase in cell index over the subsequent days. In STRlar1, the normalised cell index steadily increased throughout the observation period, whereas in STRlar2, it began to decline 4.5 days after the medium exchange. Additionally, cell index values differed between the lines: STRlar1 ranged from 0.82 to 1.74 (±0.32), while STRlar2 ranged from 0.62 to 1.23 (±0.14) during the experiment (Figure 5).

### 3.4. Ecotoxicological Response of STRlar1 Cell Line

**Figure 6:**
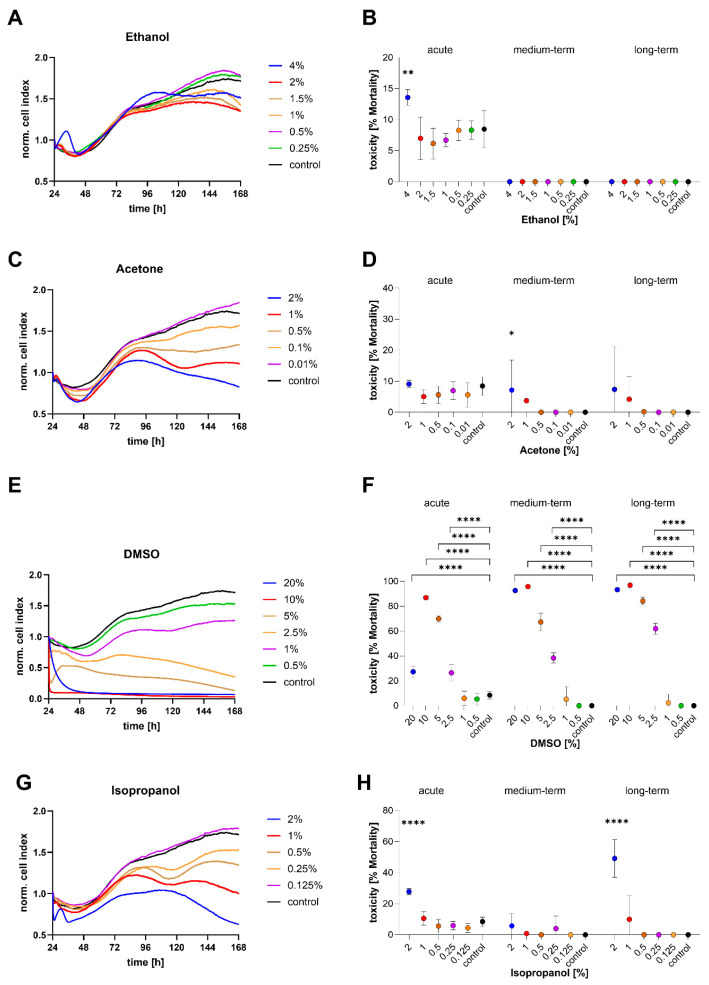
Response of STRlar1 (*Salmo trutta*) cells upon chemical exposure. Left graphs: visualisation of growth curves over time (cell index, CI) at different (**A**) ethanol concentrations (4%—blue, 2%—red, 1.5%—brown, 1%—purple, 0.5%—orange, 0.25%—green, control—black), (**C**) acetone concentrations (2%—blue, 1%—red, 0.5%—brown, 0.1%—purple, 0.01%—orange, control—black). (**E**) DMSO concentrations (20%—blue, 10%—red, 5%—brown, 2.5%—purple, 1%—orange, 0.5%—green, control—black) and (**G**) isopropanol concentrations (2%—blue, 1%—red, 0.5%—brown, 0.25%—purple, 0.125%—orange, control—black). Right graphs (**B**,**D**,**F**,**H)**: Calculated acute mortality (% CI of the first 3.5 h after exposure/medium exchange compared to the initial CI at hour 24), mid-term mortality (% CI between hour 112 and 118 h), and long-term mortality (% CI of the last 6 h of the experiment, hour 159 and 165, compared to the initial CI). *N* = 8 per experimental trial. Statistical significance (Kruskal–Wallis followed by a Dunn’s test for multiple comparisons) as indicated: * *p* < 0.05, ** *p* < 0.01, **** *p* < 0.0001).

Ethanol exposure primarily induced acute toxicity immediately after its addition, with significantly increased mortality observed only in the 4% ethanol group (13.6 ± 1.3%) compared to the control (8.5 ± 3.0%) (Figure 6A,B). No delayed mortality was detected over time, and cell growth resumed across all ethanol concentrations. However, ethanol slightly inhibited proliferation, as reflected by reduced cell impedance values at higher concentrations (Figure 6A).

In contrast, acetone exposure showed distinct time-dependent effects. While no acute toxicity was detected, a sharp decline in cell impedance occurred in the 1% and 2% acetone groups after 48 h, indicating a delayed but strong toxic response (Figure 6C). During the mid-term phase, only 2% acetone led to increased cell mortality (7.1 ± 9.7%). No further significant differences were observed at any concentration during the long-term phase when compared to the control (Figure 6D).

DMSO at concentrations of 0.5% and 1% did not cause significant differences in cell viability compared to the control (Figure 6E). Although cell impedance values were slightly lower, the overall curve progression remained similar. However, in contrast, concentrations of 2.5% and above showed clear toxic effects, with progressively declining impedance values. Notably, exposure to 10% and 20% DMSO caused immediate mortality rates of up to 86.9 ± 1.2%, which further increased to 95.8 ± 0.4% and 96.9 ± 0.5% in the mid-term and long-term phases, respectively (Figure 6F). Exposure to 5% DMSO resulted in sustained toxicity, with mortality ranging from 67.3 ± 7.1% (acute) to 84.1 ± 3.1% (long-term). Similarly, 2.5% DMSO led to a gradual increase in cell mortality over time (acute: 26.5 ± 6.5%, mid-term: 38.4 ± 4.1%, long-term: 61.9 ± 4.4%) (Figure 6F).

For isopropanol, toxic responses were only observed at the highest tested concentration of 2%. STRlar1 cells showed significant mortality in the acute phase (27.8 ± 2.1%) and in the long-term observation (49.2 ± 12.1%) compared to the control (Figure 6G,H). No significant effects were seen at lower concentrations.

### 3.5. Ecotoxicological Response of STRlar2 Cell Line

**Figure 7:**
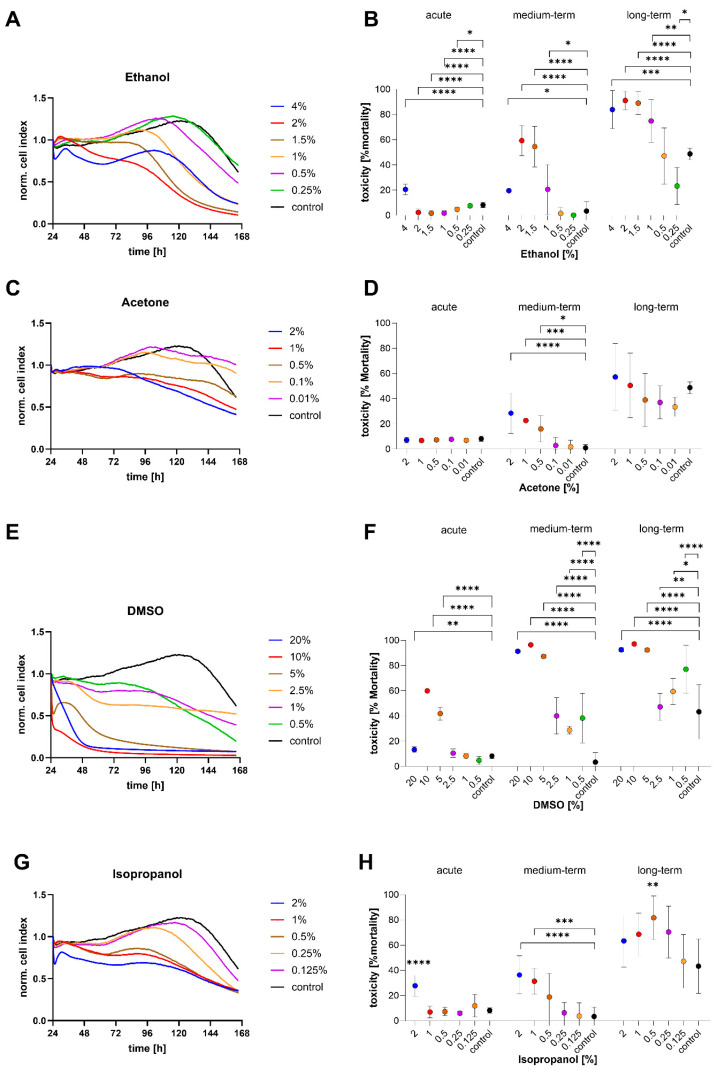
Response of STRlar2 (*Salmo trutta*) cells upon chemical exposure. Left graphs: visualisation of growth curves over time (cell index, CI) at different (**A**) ethanol concentrations (4%—blue, 2%—red, 1.5%—brown, 1%—purple, 0.5%—orange, 0.25%—green, control—black), (**C**) acetone concentrations (2%—blue, 1%—red, 0.5%—brown, 0.1%—purple, 0.01%—orange, control—black). (**E**) DMSO concentrations (20%—blue, 10%—red, 5%—brown, 2.5%—purple, 1%—orange, 0.5%—green, control—black) and (**G**) isopropanol concentrations (2%—blue, 1%—red, 0.5%—brown, 0.25%—purple, 0.125%—orange, control—black). Right graphs (**B**,**D**,**F**,**H)**: Calculated acute mortality (% CI of the first 3.5 h after exposure/medium exchange compared to the initial CI at hour 24), mid-term mortality (% CI between hour 112 and 118 h), and long-term mortality (% CI of the last 6 h of the experiment, hour 159 and 165, compared to the initial CI). *N* = 8 per experimental trial. Statistical significance (Kruskal–Wallis followed by a Dunn’s test for multiple comparisons) as indicated: * *p* < 0.05, ** *p* < 0.01, *** *p* < 0.001, **** *p* < 0.0001).

Overall, the STRlar2 cell line showed a higher sensitivity compared to STRlar1, with more pronounced responses to all tested substances. As observed in STRlar1, exposure to 4% ethanol significantly increased mortality during the acute phase (Figure 7A,B). During the proliferative growth phase, mortality rates also rose significantly at ethanol concentrations of 1%, 1.5%, 2%, and 4%, with the 2% concentration resulting in 59.3 ± 12.0% mortality. In the post-peak growth phase, ethanol concentrations from 1% to 4% led to significantly elevated mortality, with the highest rate again observed at 2% ethanol (91.1 ± 7.2%) (Figure 7A,B).

The response to acetone exposure in STRlar2 cells differed in timing. No immediate effect was observed after chemical addition, but toxic responses emerged during the mid-term (proliferative) phase (Figure 7C,D). Here, mortality increased in a concentration-dependent manner: 0.5% acetone led to 16.1 ± 10.4%, 1% to 22.7 ± 7.1%, and 2% to 28.6 ± 16.0% mortality, all significantly higher than in the control group. In the long-term phase, no significant differences in mortality were observed compared to the control (Figure 7D).

The strongest toxic response in STRlar2 cells was observed after DMSO exposure. Immediately following treatment, the three highest DMSO concentrations caused acute mortality rates of up to 60.1 ± 1.1% (Figure 7E,F). During the mid-term phase, mortality further increased at these concentrations, reaching between 87.3 ± 1.4% and 96.5 ± 0.2%. Notably, even lower concentrations of 2.5%, 1%, and 0.5% DMSO led to significantly elevated cell mortality during this observation period compared to the control. In the long-term phase, additional mortality increases were seen only at these lower DMSO concentrations, as higher concentrations had already induced near-complete cell death. Specifically, mortality at 2.5%, 1%, and 0.5% DMSO ranged from 47.3 ± 10.8% to 77.2 ± 18.9%, while the control group showed 43.4 ± 21.5% (Figure 7E,F).

The response of STRlar2 cells to isopropanol is displayed in Figure 7G, H. In the acute phase, 2% isopropanol significantly increased cell mortality to 27.8 ± 8.3%, which further rose to 36.4 ± 15.1% over time. Additionally, 1% isopropanol induced mid-term mortality of 31.4 ± 10.2%. In the long-term phase, only 0.5–isopropanol caused a significant increase in mortality (81.7 ± 17.3%), whereas all other concentrations showed similar values comparable to the control (43.4 ± 21.5%).

## 4. Discussion

In this study, two stable cell lines derived from the larval tissue of *Salmo trutta* were successfully established. Both lines demonstrated consistent proliferation over more than 40 passages, indicating their suitability for long-term use in both fundamental and applied research. Unlike mammalian cells, which typically undergo senescence due to telomere shortening [44], fish cells often spontaneously immortalise without requiring genetic modification or external intervention [13,25].

According to the Cellosaurus database [19], 80 cell lines of *Oncorhynchus mykiss* and 22 of *Salmo salar* have been registered, while only one line is listed for *S. trutta* (Accession ID: CVCL_A2SX) [45]. This significant gap highlights the need for a broader range of *S. trutta* cell models, especially given the increasing importance of this species for ecotoxicological studies. The limited availability of species-specific cell lines currently restricts research in the fields of environmental toxicology, disease modelling and developmental biology. The two newly developed larval *S. trutta* cell lines aim to fill this gap and provide valuable platforms for a wide range of applications, including toxicological testing, virology, developmental studies and environmental monitoring.

### 4.1. Comparison of Growth Behaviour

The establishment of the STRlar2 cell line proved to be more difficult than that of STRlar1. STRlar2 showed slower proliferation, higher cellular sensitivity and signs of cellular stress during the first cultivation phase. For successful establishment, cells from 12 fish larvae had to be pooled, whereas only five larvae were sufficient for STRlar1. A major difference between the two lines was the significantly larger cell size observed in early passages of STRlar2, especially when cultured in medium containing 20% FBS. As noted by Chadha et al. (2024), increased cell size during development may be an indication of cellular stress [46]. To further compare the two lines, proliferation and impedance-based growth profiles were analysed in real time over a period of 7 to 13 days. Both lines showed proliferative activity, but STRlar1 exhibited significantly higher impedance values and a longer growth phase. As the cell densities were similar at the early time points, it is unlikely that the observed differences in impedance are due to cell number. Instead, they probably reflect differences in cell–substrate interactions and binding strength. We hypothesise that the higher impedance values observed in STRlar1 are due to stronger adhesion, possibly related to the presence of lamellipodia, which play a crucial role in cell movement and substrate anchoring [47]. Lamellipodia generate traction forces at the leading edge of the cell, which are essential for forward movement and may contribute to increased electrical resistance. This is supported by studies indicating that productive cell migration requires a dynamic cycle of adhesion, formation and disassembly [48]. The impedance curve of the STRlar1 cell line continued to increase after five days of media exchange, indicating continued cell proliferation. This indicates that the cells remained metabolically active, continued to proliferate and did not reach a plateau during the observation period. In contrast, the STRlar2 cell line showed a significant decrease in impedance values after the fourth day. The decrease in impedance could be due to the lack of serum factors required for continued cell metabolism and growth, suggesting that the cells were no longer able to maintain their proliferation capacity under these conditions [49,50]. This hypothesis is supported by the fact that STRlar2 cells showed higher proliferation at the endpoint measurement, as evidenced by a significantly higher cell number compared to STRlar1. Due to the increased proliferation, more medium was consumed in the culture, which could explain the earlier plateau and subsequent drop in impedance. Without additional media exchange, factors such as acidification or toxic metabolites probably contributed to the decline in cell activity [51].

### 4.2. Reaction to Chemical Exposure

A central aim of this study was to establish cell lines of *S. trutta* (brown trout) to help reduce the use of animals in ecotoxicological research. Brown trout are widely recognised as valuable bioindicator species due to their sensitivity to environmental pollutants [8,9,10,11]. Fish cell cultures allow researchers to study the cellular effects of environmental pollutants—such as chemical pollutants, pharmaceuticals and heavy metals—without the need for live animals. Fish cell lines are already being used to study toxicological effects in vitro [15,16] and represent a promising alternative to in vivo models. However, the variety of available cell lines is still limited, especially for species that are not among the most frequently studied salmonids. Expanding the repertoire of species-specific cell lines is crucial for improving predictive power and species relevance in ecotoxicological assessments.

In this study, two cell lines of brown trout larvae were established and subsequently exposed to different chemicals to assess cellular responses and investigate within-species variability. In vitro toxicity tests on fish cells provide meaningful insights, as the concentrations that induce cell death often reflect environmentally relevant exposures that can lead to systemic toxicity or mortality at the organism level [15]. In line with previous research, this study investigated the effects of commonly used industrial solvents—ethanol, isopropanol, DMSO, and acetone—on brown trout cell lines [43]. These chemicals are frequently used in the laboratory and in industry as solvents, preservatives or cleaning agents. According to the European Chemicals Agency (ECHA) and US Environmental Protection Agency (EPA) classifications, ethanol and isopropanol are considered non-toxic at low concentrations, acetone is considered a low-hazard substance, and DMSO is generally categorised as a chemical of low concern. However, toxicity thresholds vary depending on species and exposure conditions. In fish, the species-specific LC_50_ values for DMSO are between 25 and 43 g/L [52]. These results are consistent with in vitro toxicity data of the CMAfin1 cell line at 20 °C, where higher temperatures led to increased mortality [43]. In the sturgeon cell line AOXlar7y, almost 100% of the cells died at an exposure of 5–20% [43]. Comparable toxic effects were observed in the STRlar1 cell line, while the STRlar2 cells showed a significantly higher sensitivity. STRlar2 showed significant mortality even at DMSO concentrations that are normally considered non-toxic in other fish cell models, suggesting a particular susceptibility. Although DMSO is widely used in biomedical research, particularly as a cryoprotectant and solvent in drug delivery systems [53], it becomes cytotoxic at higher concentrations. The primary toxic mechanism is thought to be disruption of the integrity of the cell membrane, leading to increased permeability, ion imbalance and ultimately loss of cellular homeostasis [54,55,56]. The marked sensitivity of STRlar2 cells to DMSO highlights the importance of interspecies variability in toxicological responses and suggests that brook trout cell lines—particularly those derived from larval tissue—need to be carefully evaluated when used in solvent-containing experimental setups. These results support the need to adapt exposure protocols for sensitive cell types and emphasise the general importance of developing multiple cell lines per species to reflect biodiversity.

Ethanol is widely used in cell culture for its antimicrobial properties, helping to reduce contamination risks [51]. However, it is also known to induce cytotoxic effects depending on concentration and exposure duration [57,58]. In the STRlar1 cell line, exposure to 4% ethanol led only to a slight increase in mortality immediately after administration. Notably, lower ethanol concentrations appeared to promote cell growth—an effect also observed by Brodowski et al. in 2021, potentially due to ethanol-induced alterations in the biophysical and biochemical properties of cell membranes [59]. In contrast, STRlar2 cell line exhibited higher sensitivity to ethanol in both a dose- and time-dependent manner, supporting the well-documented cytotoxic effects of ethanol [58,60], as well as its detrimental impact on fish development [61]. Ethanol disrupts cell membranes by solubilising lipid bilayers, leading to altered membrane structure, especially in sensitive tissues such as gills and skin. This disruption can cause osmotic imbalances and impair homeostasis. Additionally, ethanol can affect neurological processes, leading to behavioural changes, reduced motor functions, and altered sensory responses [62]. The pronounced differences in ethanol sensitivity between STRlar1 and STRlar2 suggests underlying differences in cellular composition. In the STRlar2 cell line, certain cell populations appear to be more sensitive to chemical stressors and less robust compared to those in the STRlar1 line. To better understand these variations, further characterisation is necessary—particularly through the identification of specific marker genes and proteins that define cell identity and function. Such analyses would help to clarify the cellular makeup of each line and their respective responses to environmental stressors.

Isopropanol is widely used in laboratory and industrial settings, primarily for disinfection and decontamination, due to its ability to denature proteins and induce cell death at higher concentrations [63,64]. In fish cell culture studies, isopropanol at low doses is generally considered safe, while higher levels can lead to apoptosis and reduce cell viability [43,65,66]. In the present study, both brown trout larval cell lines exhibited increased sensitivity to 2% isopropanol, with STRlar2 showing particularly elevated mortality in both the acute and long-term phases. Compared to other fish cell lines, such as those derived from maraena whitefish and Atlantic sturgeon, the brown trout lines displayed a more immediate and pronounced response [43]. These findings support the view that concentrations below 1% are [43] generally non-toxic across most fish cell models, while concentrations at or above 2% pose a significant cytotoxic risk.

Acetone is chemically comparable to isopropanol and is widely used in industry as a solvent as well as an ingredient in various consumer products [67]. Additionally, acetone is a natural metabolic by-product in many organisms [68,69]. Its impact on cell cultures was recognised early on [70]. In the present study, acetone induced only moderate increases in mortality, with time-dependent responses particularly evident in the STRlar2 cell line during the mid-term proliferation phase. These responses were comparable to those observed with isopropanol exposure.

Overall, the results support regulatory practices, such as those outlined by the International Council for Harmonisation [67], that recommend limiting residual concentrations of isopropanol and acetone in pharmaceutical and cosmetic products to below 0.5–1%. This threshold appears to effectively mitigate cytotoxic effects in vitro and ensures safety in environmentally relevant exposure scenarios.

## 5. Conclusions

The present study showed that the biodiversity of fish remains underexplored and highlights the growing relevance of in vitro models, especially those derived from larval fish, for advancing ecotoxicological and aquatic research. The newly established brown trout larval cell lines enable the assessment of direct toxic effects while eliminating confounding variables such as metabolic transformations. Their use aligns with the 3R principles, providing an ethical and scientifically robust alternative to in vivo testing. Incorporating cell lines from sensitive cold-water species, such as *Salmo trutta*, broadens the ecological relevance of in vitro toxicological models and supports the study of species adapted to specific environmental conditions. The distinct toxicological responses observed in this study—both between the two cell lines (lineage-specific) and within the species (intraspecific variability)—underline the importance of physiological diversity in toxicity assessments. Such variability provides an opportunity for more comprehensive studies (e.g., targeted gene expression analyses or experiments to inhibit signalling pathways) to further elucidate the signalling pathways responsible for chemical intolerance beyond the scope of the present analysis. These findings emphasise the need for further characterisation of fish cell lines to better understand species- and lineage-specific sensitivities and to improve the predictive power of in vitro models for environmental risk assessment.

## Data Availability

We confirm that all the data in this manuscript is original, stored with us, and available for sharing upon a reasonable request.

## Abbreviations

The following abbreviations are used in this manuscript:

3R	Replacement, Reduction and Refinement
DMSO	Dimethyl sulfoxide
ECHA	European Chemicals Agency
EDTA	Ethylenediaminetetraacetic acid
EPA	US Environmental Protection Agency
FBS	Foetal Bovine Serum
IUCN	International Union for Conservation of Nature and Natural Resources
L-15	Leibovitz-15
OECD	Organisation for Economic Co-operation and Development
P/S	Penicillin/streptomycin
PBS	Dulbecco’s Phosphate-Buffered Saline
REACH	Registration, Evaluation, Authorization and Restriction of Chemicals
TierSchG	Tierschutzgesetz
TPP	Techno Plastic Products AG
